# Distribution of HLA-DRB1 Alleles in Patients With Antiphospholipid Syndrome and Their Association With Antiphospholipid Antibodies Presence and Damage Indexes

**DOI:** 10.1155/jimr/2827348

**Published:** 2025-03-24

**Authors:** Ewa Haladyj, Barbara Stypinska, Agata Matusiewicz, Marzena Olesinska, Agnieszka Paradowska-Gorycka

**Affiliations:** ^1^Eli Lilly and Company, Indianapolis, USA; ^2^Department of Molecular Biology, National Institute of Geriatrics, Rheumatology and Rehabilitation, Warsaw 02-637, Poland; ^3^Department of Connective Tissue Diseases, National Institute of Geriatrics, Rheumatology and Rehabilitation, Warsaw 02-637, Poland

**Keywords:** antiphospholipid syndrome (APS), human leukocyte antigen (HLA), systemic lupus erythematosus (SLE)

## Abstract

Antiphospholipid syndrome (APS) is frequently coexisting with systemic lupus erythematosus (SLE) and the knowledge on its genetic background is essential. The objective of this work was to assess distribution of human leukocyte antigen (HLA)-DRB1 alleles in patients with APS with or without SLE in the context of Polish population data. The study was performed in a group of 112 patients with APS and healthy subjects to assess the distribution of HLA-DRB1 alleles in patients with APS and their association with clinical characteristics of patients with APS—antiphospholipid antibodies (aPLs) presence and disease activity/damage indexes. The distribution of HLA-DRB1 alleles showed significant differences between patients with APS and healthy subjects. Allelic variant HLA-DRB1*⁣*^*∗*^1.15 was identified as risk alleles for APS observed in patients with APS (odds ratio (OR): 2.06 (1.27, 3.23), *p*=0, 004), while HLA-DRB1*⁣*^*∗*^1.07 showed significant protective association (OR: 0.37 (0.14–0.76), *p*=0, 006). In subgroup of patients with coexisting SLE allelic variants above were not identified as risk or protective, while protective association was described for HLA-DRB1*⁣*^*∗*^01, but not for patients in primary APS group. Presence of antibodies anti-*β*_2_-glycoprotein-I (a*β*_2_GPI) IgA and against domain 1, anti-phosphatidylserine/prothrombin (aPS/PT) and anticardiolipin antibody (aCL) IgA all the antibodies which were negatively associated with HLA-DRB1*⁣*^*∗*^15.01 carriers, what was reported for the first time may be suitable in discussion about value of these antibodies in practice and scientific research. This study clearly shows that primary APS has a distinct HLA-DRB1 associations as compared with SLE with a strong positive association with HLA-DRB1*⁣*^*∗*^15.01 allele and a protective association with HLA-DRB1*⁣*^*∗*^07.01.

## 1. Introduction

Antiphospholipid syndrome (APS) is a systemic autoimmune disorder manifesting clinically with thrombotic, nonthrombotic, and obstetric complications in the persistent presence of antiphospholipid antibodies (aPLs) [1]. Anticardiolipin antibodies (aCLs), anti-*β*_2_-glycoprotein-I (a*β*_2_GPI) antibodies, and lupus anticoagulant (LAC) functional coagulation assay are a part of classification criteria for APS and are widely recognized by clinicians and researchers. However, there is much more aPL, among them anti-phosphatidylserine/prothrombin (aPS/PT) complex antibodies, a*β*_2_GPI antibodies against domain I (a*β*_2_GPIDI) and anti IgA class (a*β*_2_GPI IgA) were also described as highly associated with APS symptoms [2–4]. In the latest ACR–EULAR APS classification criteria above aPL have been shown promising as prognostic markers and deserve continued standardization and studies to determine their diagnostic roles [1].

APS, in almost half of the cases, appears as an isolated disorder known as primary APS, however, an underlying systemic autoimmune disease, commonly lupus, is frequently coexisting [5]. Beyond the presence of an underlying autoimmune disorder, no clinical differences have been reported between the primary APS and associated with other autoimmune diseases.

Pathogenesis of APS similarly to many other autoimmune diseases base on diverse antigenic stimulation of predisposed patients. The most convincing proofs of genetic predisposition to APS was described in familiar clustering cases, in patients with high titers of aPL in the serum sharing the same descent of patients, animal models (mice), and association with human leukocyte antigen (HLA) alleles [6].

Among the most relevant and studied genetic factors for ADs are genes located in the major histocompatibility complex (MHC) and loci from HLA class I and class II are the most relevant and studied genetic factors for autoimmune diseases. Particularly, HLA class II genes has been associated with systemic lupus erythematosus (SLE) or APS. Alleles differ from one population to another, and also within the same population, and finally interplay between genetic effect of HLA and environmental factors associated with autoimmune diseases [2].

The objective of the study was to assess the distribution of HLA-DRB1 alleles in patients with APS and their association with clinical characteristics of patients with APS–aPL presence and disease activity/damage indexes. The study was performed in a group of primary APS and coexisting with SLE.

## 2. Patients and Methods

### 2.1. Subjects

The research was conducted in a group of patients hospitalized in the years 2009–2015 at the Clinic and Polyclinic of Systemic Connective Tissue Diseases of the Institute of Rheumatology in Warsaw. The study involved 112 patients with APS diagnosed according to the Sydney consensus criteria (2006) [7]. Patients were divided into two groups of similar size based on the presence of a concomitant disease—SLE, diagnosed based on the classification criteria according to SLICC (2012) [8]. Data from the history and physical examination as well as demographic data were collected in the patient's examination protocol and then transferred to a computer database. Genetic analyses indicate that the Polish population is relatively homogeneous, with slight regional differences. Studies on mitochondrial DNA and Y-chromosome variability show low levels of genetic differentiation within Poland, suggesting a high degree of genetic homogeneity [9, 10]. Typing of HLA-DRB1 alleles in Polish healthy volunteers was performed by Schmidt et al. [11]. In our study, the alleles of HLA-DRB1 were assessed in all cohorts and compared with the data on frequencies of DRB1 alleles (41,306 alleles) in Polish donors derived from Schmidt et al. [11] study.

Patients' medical history, physical examination, and additional tests resulting from the specific clinical picture of APS and SLE and the involvement of internal organs observed in individual patients were performed. All patients underwent the following analytical tests at the Central Clinical Laboratory of NIGRiR, in accordance with the procedures recommended by the manufacturers: peripheral blood count—using the XT 2000 I analyzer (CBC-6DIF); biochemical tests: concentration of C-reactive protein (CRP), glucose, urea, creatinine, lipid profile, alanine transaminase (ALT) and aspartate transaminase (AST), creatine kinase (CK), and lactate dehydrogenase (LDH)—determinations were made on biochemical analyzers using Vitros 350 thin film technology; proteinogram—by capillary electrophoresis, using the Capilaris Sebia analyzer; general urine test—URISYS analyzer; Biernacki's test (ESR)—Sedi System red blood cell sedimentation analyzer, (ref.range up to 12 mm/h); high-sensitivity CRP (hsCRP), complement components C3 and C4—the nephelometric method using IMMAGE 800; using the nephelometric method, using the IMMAGE 800 analyzer; LAC—a screening test and a confirmation test using excess phospholipids in the hexagonal phase and Russell's viper venom, using the TOP 300 analyzer (reagents from InsSLEmentation Laboratory); aCLs in the IgM and IgG classes enzyme-linked immunosorbent assay (ELISA)), according to the modified Gharavi method and our own modification [12] (reagents from Sigma, USA), antibodies against *β*2 glycoprotein I in the IgM, IgG, IgA, and D1; aCL IgA—chemiluminescence test QuantaLite B2 GPI IgM and IgG ELISA Kit by Inova Diagnostics on the BioFlash analyzer by Werfen, antinuclear antibodies—indirect immunofluorescence test using Hep-2 cells from Euroimmun Polska; panel of autoantibodies for the following antigens: U1-RNP-70, U1-RNP-A, U1-RNP-C, Sm-B, Sm-D, Ro/SSA 60kD, Ro/SSA 52kD, La/SSB, RibP, PCNA, CENPB, Scl-70, Jo-1, dsDNA, and histones—DOT-blot method, recomLine ANA/ENA IgG test from Mikrogen Diagnostik; antibodies antiphosphatodyloserine IgM, IgG, IgA, and antiprothrombin IgG, IgM by ELISA Kit by Inova Diagnostics on the BioFlash analyzer by Werfen.

#### 2.1.1. DNA Extraction

Genomic DNA was extracted from whole blood collected into EDTA tubes from patients with APS using the standard guanidine isothiocynate (GTC) extraction method and/or the QIAamp DNA Blood Mini Kit (Qiagen). DNA purity and concentration were determined by spectrophotometric measurement of absorbance at 260 and 280 nm.

#### 2.1.2. Determination of HLA-DRB1 Alleles

To define the HLA-DRB1 alleles, DNA sequences for exon 2 of the DRB1 locus were typed using polymerase chain reaction-sequence-based typing (PCR-SBT). Gene-specific oligonucleotide primer mix [13] was used. The primer mix contained each of seven 5′-amplification primers and the single 3′-amplification primer. Table 1 from the previously published aricle [13] provided information about the primer sequences and the length of the PCR products. Amplification reaction was performed in a total amount of 20 µL PCR mixture, which contained 2 µL (40 ng/µL) of genomic DNA, 2 µL of 10× PCR buffer (with 15 mmol MgCl_2_), 0.2 µL of 10 mM dNTP mix (Qiagen), 1.2 µL of each primer (10 µmol/µL), 0.1 µL (1 U) of HotStart Taq Polymerase (Qiagen), and 14.5 µL of water. The PCR reaction was performed using G-STORM (Labtech) and/or ATC401 (CLP) instruments with the following conditions: one cycle: 94°C for 5 min; three cycles: 94°C for 30 s, 68°C for 30 s, and 72°C for 30 s; three cycles: 94°C for 30 s, 65°C for 30 s, and 72°C for 30 s; 30 cycles: 94°C for 30 s, 62°C for 30 s, and 72°C for 30 s, and finally 72°C for 7 min. PCR products were identified on 1.5% agarose gel and then purified by a PCR cleanup reagent. In case of positive results, the nucleotide sequences of PCR products were directly sequenced by Sanger method. DNA sequencing of both strands was performed using M13 primers.

### 2.2. Statistical Analysis

Chi-squared test or Monte Carlo test with 2000 replicates (Hope, 1968) were used for estimating association between the frequencies. Number of observations, percents and the odds ratios (ORs) with 95% confidence intervals (CIs) were estimated. Continuous variables were compared using *t* test or Mann–Whitney *U* test. Mean values and standard deviations were presented. Differences were considered statistically significant if *p*-value was <0.05. All calculations, tables, and figures were conducted in R program. (R Core Team (2021). R: A language and environment for statistical computing. R Foundation for Statistical Computing, Vienna, Austria.)

## 3. Results

The study included 112 patients with APS, including 57 (50.9%) with primary APS (APS) and 55 (49.1%) with concomitant SLE (APS/SLE group). Both groups were dominated by women. The average age of patients at the time of the study was 48 years. There were no differences in age, gender, or disease duration between the groups. The incidence of total thrombosis and obstetric manifestations did not differ between the APS and APS/SLE groups, but venous thrombosis was more frequently observed in the APS/SLE group (Table 1). Other organ-specific symptoms occurred with similar frequency in both groups. Most often, they concerned the nervous system and skin.

Among aPL, LAC was the most frequent (81,25%) and the least aCL IgM (9.82%). Triple positivity of aPL was similar across groups. Antinuclear, anti-dsDNA and antiphosphatyloserine IgG antibodies were more frequent in SLE/APS group (Table 2).

### 3.1. Distribution of HLA-DRB1 Alleles in Patients With APS

Thirty-three DRB1 alleles are present in at least 0.1% in Polish population from well documented 410 functional HLA-DRB1 alleles [14]. In present study HLA-DRB1 7 alleles were assessed in APS patient group and compared with the data on frequencies of DRB1 alleles (41,306 alleles) in Polish donors derived from Schmidt et al. [11]. The distributions of HLA-DRB1 allele frequencies among patients and controls, as well as their associations with the risk of APS are shown in Tables 3–6.

The distribution of two HLA-DRB1 alleles showed significant differences between patients with APS and healthy subjects (Table 3). Allelic variant identified as risk alleles for APS is HLA-DRB1*⁣*^*∗*^1.15 (OR: 2.06 (1.27, 3.23), *p*=0, 004), what was observed in whole group and APS group—but not for patients with coexisting SLE. Similarly, HLA-DRB1*⁣*^*∗*^1.07 (OR: 0.37 (0.14–0.76), *p*=0, 006) showed significant protective association for all patients and those with primary APS, but not in SLE/APS group. At the same time in all group and SLE/APS protective association was described for HLA-DRB1*⁣*^*∗*^01, but not for patients in APS group.

When comparing groups APS and SLE/APS group, allele HLA-DRB1*⁣*^*∗*^07 was more frequent in APS/SLE group (OR: 4.09 (1.44, 4.009), *p*=0, 012).

### 3.2. The Association of HLA-DRB1 Alleles With Clinical Characteristics of Patients With APS

**Figure 1:**
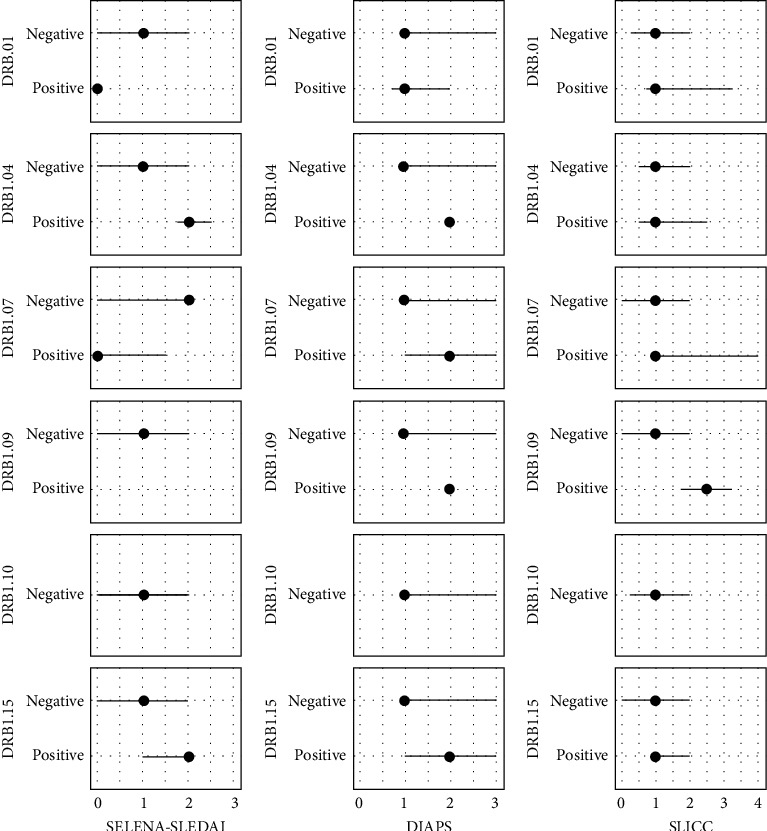
(A–C) Association of selected human leukocyte antigen (HLA)-DRB1 alleles with the degree of systemic lupus erythematosus (SLE) activity measured by the SLEDAI scale (A; 2*n* = 54) or damage due to antiphospholipid syndrome (APS) and SLE according to DIAPS (B; 2*n* = 158) and SLICC (C; 2*n* = 158). SLEDAI was available only for SLE/APS group (2 *n* = 54). Dots indicate medians and lines show 1^st^ and 3^rd^ quartile. All *p* values > 0.05.

There was no association of the occurrence of selected alleles with damage in the course of APS and SLE according to DIAPS and SLICC (Figure 1). However, it should be noted that the number of observations for individual alleles is very small, and the analysis does not allow for a correct assessment of such a relationship.

Because we found possible association between HLA-DRB1*⁣*^*∗*^15.01 and HLA-DRB1*⁣*^*∗*^1.07 alleles with susceptibility to APS, we decided to analyze the potential association between all these HLA-DRB1 alleles and disease activity variables as well as aPL presence (Tables 7 and 8).

We found significant negative associations between HLA-DRB1*⁣*^*∗*^15.01 and presence of aCL IgA, a*β*_2_GPI IgA and against D1, LAC, and aPS/PT IgG (Table 7).

No significant associations were observed between thrombosis or obstetric manifestations presence (data not shown).

## 4. Discussion

In the present manuscript, we identified HLA-DRB1*⁣*^*∗*^1.15 as a risk allele for patients with primary APS and HLA-DRB1*⁣*^*∗*^1.07 as protective, what was not confirmed for SLE/APS. At the same time in all group and SLE/APS protective association was described for HLA-DRB1*⁣*^*∗*^01, but not for patients in APS group.

SLE and APS are autoimmune diseases which develop in individuals with genetic predisposition. Data on APS population is coming mainly form studies with small number of participants, while knowledge on SLE predisposed patients is more extensive. Among various HLA-DR and HLA-DQ alleles published as associated with SLE, HLA- DR2 (*⁣*^*∗*^1501), and -DR3 (*⁣*^*∗*^0301) are the strongest [15, 16]. An extensive study with approximately 300 families found three class II-containing SLE-risk haplotypes, namely, DRB1*⁣*^*∗*^1501(DR2)/DQB1*⁣*^*∗*^0602, DRB1*⁣*^*∗*^0301(DR3)/DQB1*⁣*^*∗*^0201, and DRB1*⁣*^*∗*^0801(DR8)/DQB1*⁣*^*∗*^0402, the last being the least frequent [17]. In the presented work, we identified protective association for HLA-DRB1*⁣*^*∗*^01 not identified before.

On the other hand, HLA-DRB1*⁣*^*∗*^04 (DR4), -DRB1*⁣*^*∗*^07 (DR7), -DRw53, -DRB1*⁣*^*∗*^09(DR9; in a Japanese population), and among HLA-DQB1 alleles the DQB1*⁣*^*∗*^0301(DQ7) or the DQB1*⁣*^*∗*^0302(DQ8) were significantly associated with APS or with different aPLs [18–20].

This work identified HLA-DRB1*⁣*^*∗*^1.15 as risk allele and HLA-DRB1*⁣*^*∗*^1.07 as protective for patients with primary APS in Polish population. In Hungarian study HLA-DRB1*⁣*^*∗*^15 allele there were no significant differences between the SLE patients with or without APS. No significant differences in the occurrence of DRB1*⁣*^*∗*^07 alleles among the four groups, what may suggest different genetic background of pts with APS in central and eastern Europe [21]. At the same time in the study of British, Caucasian population of patients with APS [22] HLA-DRB1*⁣*^*∗*^1.07 was identified as correlating with APS, thrombosis, pregnancy loss, and a*β*_2_GPI antibodies presence—more pronounced in whole group than primary APS. In the present study HLA-DRB1*⁣*^*∗*^1.07 did not correlated with a*β*_2_GPI. The difference between these studies may come from small sample sizes and mixed population of primary APS and APS accompanied by SLE.

So far, an association between HLA-DRB1*⁣*^*∗*^1.302 and primary APS appears to be independent of the association in the HLA-DRA locus [23, 24]. However, we did not find these loci in the analyzed group.

We report for the first time association of antibodies a*β*_2_GPI IgA and against domain 1 with HLA-DRB1*⁣*^*∗*^15.01. So far, a*β*_2_GPI showed positive association with DRB1*⁣*^*∗*^04/DQB1*⁣*^*∗*^0302 [18, 25] and DRB1*⁣*^*∗*^1302/DQB1*⁣*^*∗*^0604/5/6/7/9 was also significantly increased in Caucasian and black patients [22, 25]. Other antibodies described in this paper as associated with HLA-DRB1*⁣*^*∗*^15.01 are aPS/PT and aCL IgA; so far, alleles most frequently associated with the presence of aCL were DRB1*⁣*^*∗*^04, DRB1*⁣*^*∗*^07, DRw53, DQA1*⁣*^*∗*^0201, DQA1*⁣*^*∗*^0301, and DQB1*⁣*^*∗*^0302.10–12 [18–20].

All these antibodies, according to the current literature, are not routinely tested in APS. However, they are frequently observed in APS in patients without thrombosis or pregnancy loss history. At the same time, isotype characterization is also critical since IgG aPL are more diagnostic/prognostic than IgA or IgM. There is evidence that a*β*_2_GPIDI antibodies display a stronger diagnostic/prognostic value [26].

This study has some limitations, like small sample size involving different patient populations is required to validate the results. However sample size for disease like APS is quite large, the presence of HLA-DRB-alleles were limited, so was also the possibility to make comparisons between groups. Differences in gender between APS and SLE/APS groups were small, but still may influence the results. Then, only some HLA-DRB1 allele was tested due to limited funding. We used multiple comparisons and many associations were not strong. Further studies on larger patient cohorts and including larger number of HLA alleles would be desirable to reach sufficient statistical power for these observations.

## 5. Conclusions

This study clearly shows that primary APS has a distinct HLA-DRB1 associations as compared with SLE with a strong positive association with HLA-DRB1*⁣*^*∗*^15.01 allele. In addition, it identifies HLA-DRB1*⁣*^*∗*^07.01 allele as a protective allele against APS. Association of antibodies a*β*_2_GPI IgA and against domain 1, aPS/PT and aCL IgA with HLA-DRB1*⁣*^*∗*^15.01 reported for the first time may be suitable in discussion about value of these antibodies in practice and scientific research. We propose that these HLA-DRB1 alleles may prove to be useful genetic markers for APS patients.

## Figures and Tables

**Table 1:** Baseline patients characteristics.

	All (*N* = 112)	APS (*N* = 57)	SLE/APS (*N* = 55)	*p*-Value*⁣*^*∗*^
Women, *n* (%)	97 (86.61%)	46 (80.70%)	51 (92.73%)	Ns
Age, mean ± SD	48.29 ± 13.69	46.91 ± 14.26	49.71 ± 13.05	Ns
APS duration, months, median (1stQ, 3rdQ)	72.00 (24.00, 144.00)	48.00 (13.00, 120.00)	80.00 (33.50, 156.00)	Ns
Thrombosis overall, *n* (%)	92 (82.14%)	45 (78.95%)	47 (85.45%)	Ns
Venous thrombosis, *n* (%)	58 (51.79%)	23 (40.35%)	35 (63.64%)	0.023
Arterial thrombosis, *n* (%)	49 (43.75%)	26 (45.61%)	23 (41.82%)	Ns
Obstetric APS, *n* (%)	39 (34.82%)	19 (33.33%)	20 (36.36%)	Ns

Abbreviations: APS, antiphospholipid syndrome; SLE, systemic lupus erythematosus.

*⁣* ^*∗*^Comparison between APS and APS/SLE groups.

**Table 2:** aPL characteristics.

Antibodies	All (*N* = 112)	APS (*N* = 57)	SLE/APS (*N* = 55)	*p*-Value*⁣*^*∗*^
aPL, n (%)				
aCL IgG+	22 (19.64%)	13 (22.81%)	9 (16.36%)	Ns
aCL IgM+	11 (9.82%)	6 (10.53%)	5 (9.09%)	Ns
a*β*_2_GPI IgG+	74 (66.07%)	37 (64.91%)	37 (67.27%)	Ns
a*β*_2_GPI IgM+	16 (14.29%)	9 (15.79%)	7 (12.73%)	Ns
LAC+	91 (81.25%)	47 (82.46%)	44 (80.00%)	Ns
Triple positive (a*β*_2_GPI + aCL+LAC)	21 (18.75%)	11 (19.30%)	10 (18.18%)	Ns
a*β*_2_GPI IgA	23/101 (22.77%)	7/53 (13.21%)	16/48 (33.33%)	0.030
a*β*_2_GPI D1	41/101 (40.59%)	18/53 (33.96%)	23/48 (47.92%)	Ns
aCL IgA	21/101 (20.79%)	7/53 (13.21%)	14/48 (29.17%)	Ns
aPS/PT IgM	67/101 (66.34%)	37/53 (69.81%)	30/48 (62.50%)	Ns
aPS/PT IgG	50/101 (49.50%)	22/53 (41.51%)	28/48 (58.33%)	Ns
aPS/PT IgA	6/101 (5.94%)	1/53 (1.89%)	5/48 (10.42%)	Ns
aPS IgM	35/101 (34.65%)	20/53 (37.74%)	15/48 (31.25%)	Ns
aPS IgG	52/101 (51.49%)	20/53 (37.74%)	32/48 (66.67%)	0.007
aPS IgA	6/101 (5.94%)	1/53 (1.89%)	5/48 (10.42%)	Ns
ANA+	83 (74.11%)	37 (64.91%)	46 (83.64%)	0.041
anti-dsDNA+	17 (15.18%)	5 (8.77%)	12 (21.82%)	0.005

Abbreviations: aCL, anticardiolipin antibody; aPL, antiphospholipid antibody; APS, antiphospholipid syndrome; aPS, anti-phosphatidylserine; LAC, lupus anticoagulant; PT, prothrombin; SLE, systemic lupus erythematosus.

*⁣* ^*∗*^Comparison between APS and APS/SLE groups.

**Table 3:** Distribution of HLA-DRB1 alleles in patients with APS vs. controls.

HLA-DRB1	Control (2*n* = 41,306)	APS (2*n* = 104)	OR	*p* value
HLA-DRB*⁣*^*∗*^01	4177 (10.11%)	7 (6.73%)	0.65 (0.27, 1.31)	Ns
HLA-DRB1*⁣*^*∗*^04	2206 (5.34%)	7 (6.73%)	1.30 (0.55, 2.62)	Ns
HLA-DRB1*⁣*^*∗*^07	5984 (14.49%)	6 (5.77%)	0.37 (0.14, 0.76)	0.006
HLA-DRB1*⁣*^*∗*^09	326 (0.79%)	2 (1.92%)	2.65 (0.41, 8,37)	Ns
HLA-DRB1*⁣*^*∗*^10	383 (0.93%)	0 (0%)	—	NA
HLA-DRB1*⁣*^*∗*^15	5021 (12.16%)	23 (22.12%)	2.06 (1.27, 3.23)	0.004

Abbreviations: APS, antiphospholipid syndrome; HLA, human leukocyte antigen; OR, odds ratio.

**Table 4:** Distribution of HLA-DRB1 alleles between APS and APS/SLE patients.

HLA-DRB1	APS/SLE (2*n* = 54)	APS (2*n* = 104)	OR	*p* value
HLA-DRB*⁣*^*∗*^01	1 (1.85%)	7 (6.73%)	0.29 (0.011, 1.765)	Ns
HLA-DRB1*⁣*^*∗*^04	4 (7.41%)	7 (6.73%)	1.12 (0.273, 4.009)	Ns
HLA-DRB1*⁣*^*∗*^07	11 (20.37%)	6 (5.77%)	4.09 (1.440, 12.808)	0.012
HLA-DRB1*⁣*^*∗*^09	0 (0%)	2 (1.92%)	—	NA
HLA-DRB1*⁣*^*∗*^10	0 (0%)	0 (0%)	—	NA
HLA-DRB1*⁣*^*∗*^15	7 (12.96%)	23 (22.12%)	0.53 (0.196, 1.292)	Ns

Abbreviations: APS, antiphospholipid syndrome; HLA, human leukocyte antigen; OR, odds ratio; SLE, systemic lupus erythematosus.

**Table 5:** Association between HLA-DRB1*⁣*^*∗*^15.01 and aPL antibodies.

Antibodies	HLA-DRB1*⁣*^*∗*^15.01		*p*-Value
	Negative (*N* = 128) *n* (%)	Positive (*N* = 30) *n* (%)	
aCL IgG +	29 (22.66%)	7 (23.33%)	1.00
aCL IgM +	13 (10.16%)	3 (10.00%)	1.00
aCL IgA +	26/123 (21.14%)	0/29 (0.00%)	0.012
a*β*_2_GPI IgG +	87 (67.97%)	19 (63.33%)	0.787
a*β*_2_GPI IgM +	18 (14.06%)	8 (26.67%)	0.096
a*β*_2_GPI IgA +	28/123 (22.76%)	0/29 (0.00%)	0.0099
a*β*_2_GPI D1 +	62/123 (50.41%)	4/29 (13.79%)	<0.001
LAC +	114 (89.06%)	20 (66.67%)	0.004
aPS PT IgG +	65/123 (52.85%)	11/29 (37.93%)	0.216
aPS PT IgM +	87/123 (70.73%)	17/29 (58.62%)	0.298
aPS PT IgA +	6/123 (4.88%)	0/29 (0.00%)	0.360
aPS IgG+	69/123 (56.10%)	7/29 (24.14%)	0.0039
aPS IgM +	44/123 (35.77%)	10/29 (34.48%)	1.00
aPS IgA +	6/123 (4.88%)	0/29 (0.00%)	0.367

Abbreviations: aCL, anticardiolipin antibody; aPL, antiphospholipid antibody; aPS, anti-phosphatidylserine; a*β*_2_GPI, anti-*β*_2_-glycoprotein-I; HLA, human leukocyte antigen; LAC, lupus anticoagulant; PT, prothrombin.

**Table 6:** Association between HLA-DRB1*⁣*^*∗*^07.01 and aPL antibodies.

Antibodies	HLA-DRB1*⁣*^*∗*^07.01		*p*-Value
	Negative (*N* = 141) *n* (%)	Positive (*N* = 17) *n* (%)	
aCL IgG +	35 (24.82%)	1 (5.88%)	0.146
aCL IgM +	14 (9.93%)	2 (11.76%)	1.000
aCL IgA +	22/135 (16.30%)	4 (23.53%)	0.516
a*β*_2_GPI IgG +	95 (67.38%)	11 (64.71%)	1.000
a*β*_2_GPI IgM +	26 (18.44%)	0 (0.00%)	0.088
a*β*_2_GPI IgA +	23/135 (17.04%)	5 (29.41%)	0.364
a*β*_2_GPI D1 +	57/135 (42.22%)	9 (52.94%)	0.561
LAC +	118 (83.69%)	16 (94.12%)	0.311
aPS PT IgG +	69/135 (51.11%)	7 (41.18%)	0.607
aPS PT IgM +	90/135 (66.67%)	14 (82.35%)	0.301
aPS PT IgA +	6/135 (4.44%)	0 (0.00%)	0.622
aPS IgG+	66/135 (48.89%)	10 (58.82%)	0.607
aPS IgM +	47/135 (34.81%)	7 (41.18%)	0.804
aPS IgA +	6/135 (4.44%)	0 (0.00%)	0.619

Abbreviations: aCL, anticardiolipin antibody; aPL, antiphospholipid antibody; aPS, anti-phosphatidylserine; a*β*_2_GPI, anti-*β*_2_-glycoprotein-I; HLA, human leukocyte antigen; LAC, lupus anticoagulant; PT, prothrombin.

**Table 7:** Distribution of HLA-DRB1 alleles in patients with APS/SLE vs. controls.

HLA-DRB1	Control (2*n* = 41,306)	APS/SLE (2*n* = 54)	OR	*p* value
HLA-DRB*⁣*^*∗*^01	4177 (10.11%)	1 (1.85%)	0.19 (0.008, 0.857)	0.04
HLA-DRB1*⁣*^*∗*^04	2206 (5.34%)	4 (7.41%)	1.47 (0.435, 3.610)	Ns
HLA-DRB1*⁣*^*∗*^07	5984 (14.49%)	11 (20.37%)	1.52 (0.744, 2.862)	Ns
HLA-DRB1*⁣*^*∗*^09	326 (0.79%)	0 (0%)	—	NA
HLA-DRB1*⁣*^*∗*^10	383 (0.93%)	0 (0%)	—	NA
HLA-DRB1*⁣*^*∗*^15	5021 (12.16%)	7 (12.96%)	1.098 (0.449, 2.279)	Ns

Abbreviations: APS, antiphospholipid syndrome; HLA, human leukocyte antigen; OR, odds ratio; SLE, systemic lupus erythematosus.

**Table 8:** Distribution of HLA-DRB1 alleles in patients vs. controls.

HLA-DRB1	Control (2*n* = 41,306)	All patients (2*n* = 158)	OR	*p* value
HLA-DRB*⁣*^*∗*^01	4177 (10.11%)	8 (5.06%)	0.48 (0.216, 0.922)	0.035
HLA-DRB1*⁣*^*∗*^04	2206 (5.34%)	11 (6.96%)	1.34 (0.683, 2.37)	Ns
HLA-DRB1*⁣*^*∗*^07	5984 (14.49%)	17 (10.76%)	0.72 (0.417, 1.154)	Ns
HLA-DRB1*⁣*^*∗*^09	326 (0.79%)	2 (1.27%)	1.73 (0.267, 5.435)	Ns
HLA-DRB1*⁣*^*∗*^10	383 (0.93%)	0 (0%)	—	NA
HLA-DRB1*⁣*^*∗*^15	5021 (12.16%)	30 (18.99%)	1.70 (1.121, 2.50)	0.009

Abbreviations: HLA, human leukocyte antigen; OR, odds ratio.

## Data Availability

The data that support the findings of this study are available on request from the corresponding author (Ewa Haladyj).
